# Supplementary figures and images for: Homeostatic regulation of rapid eye movement sleep by the preoptic area of the hypothalamus
Source: eLife. 2024 Jun 17;12:RP92095. doi: 10.7554/eLife.92095 (PMC11182646; doi:10.7554/eLife.92095)

Supplementary File 1. Statistical analysis


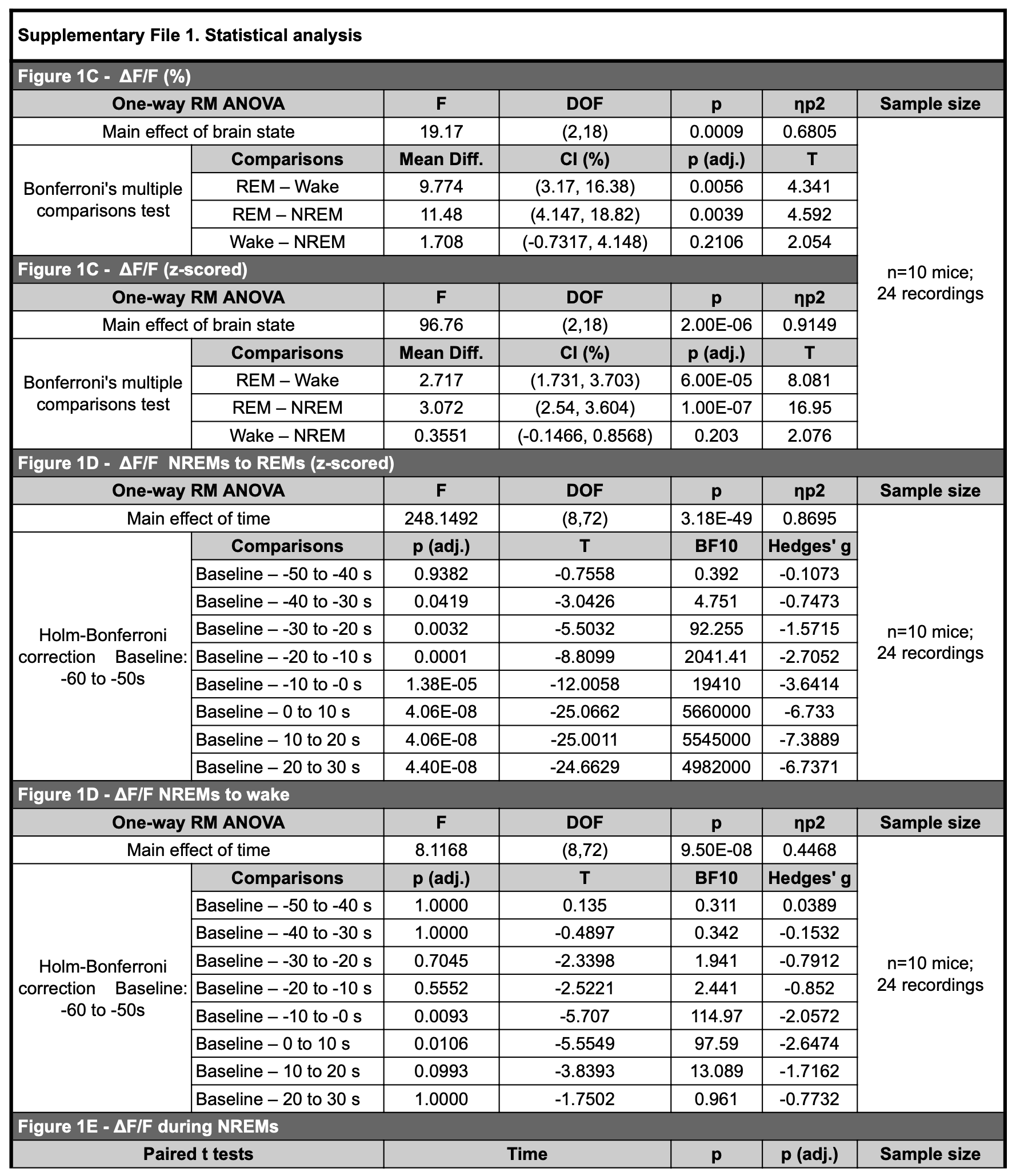


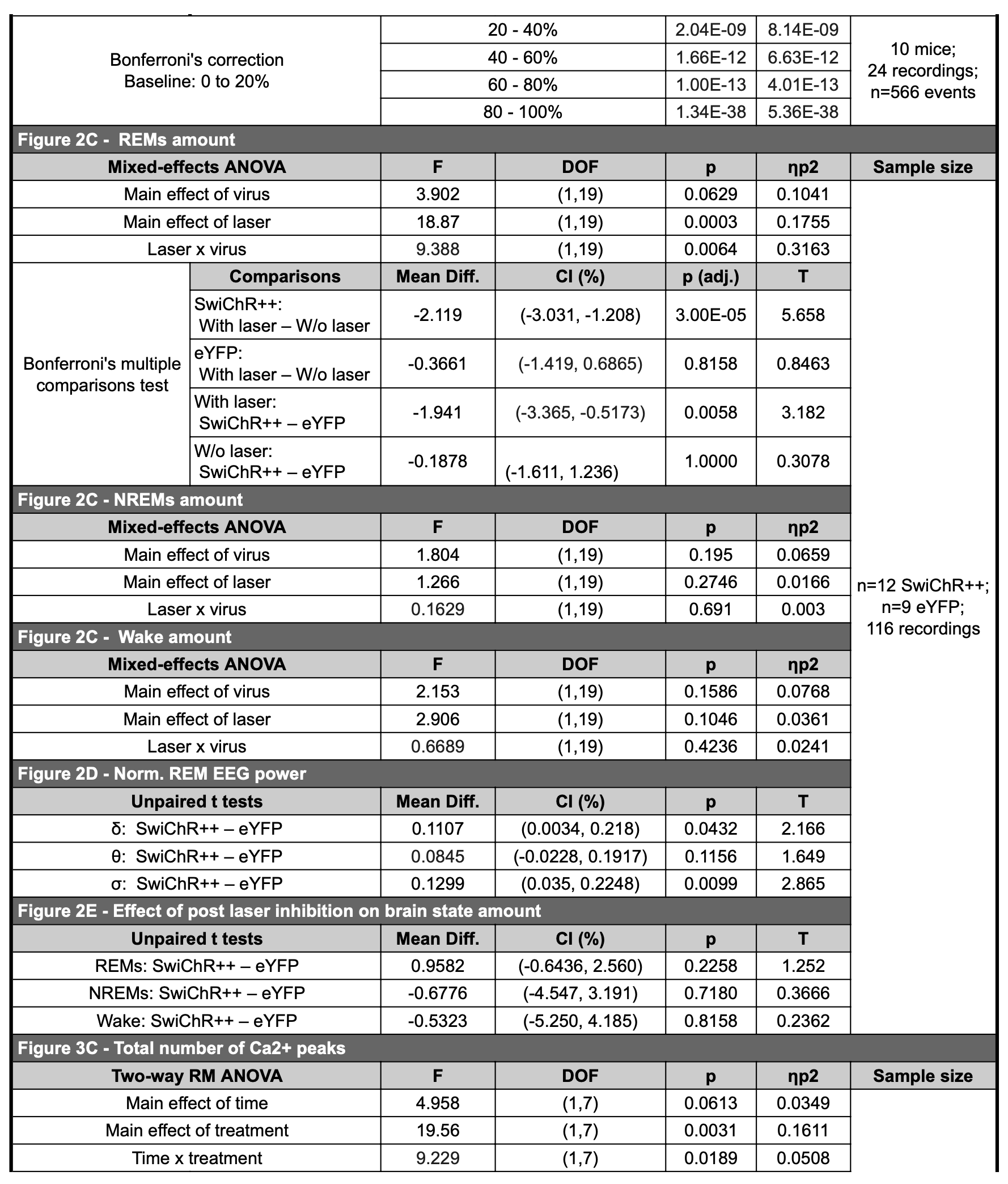


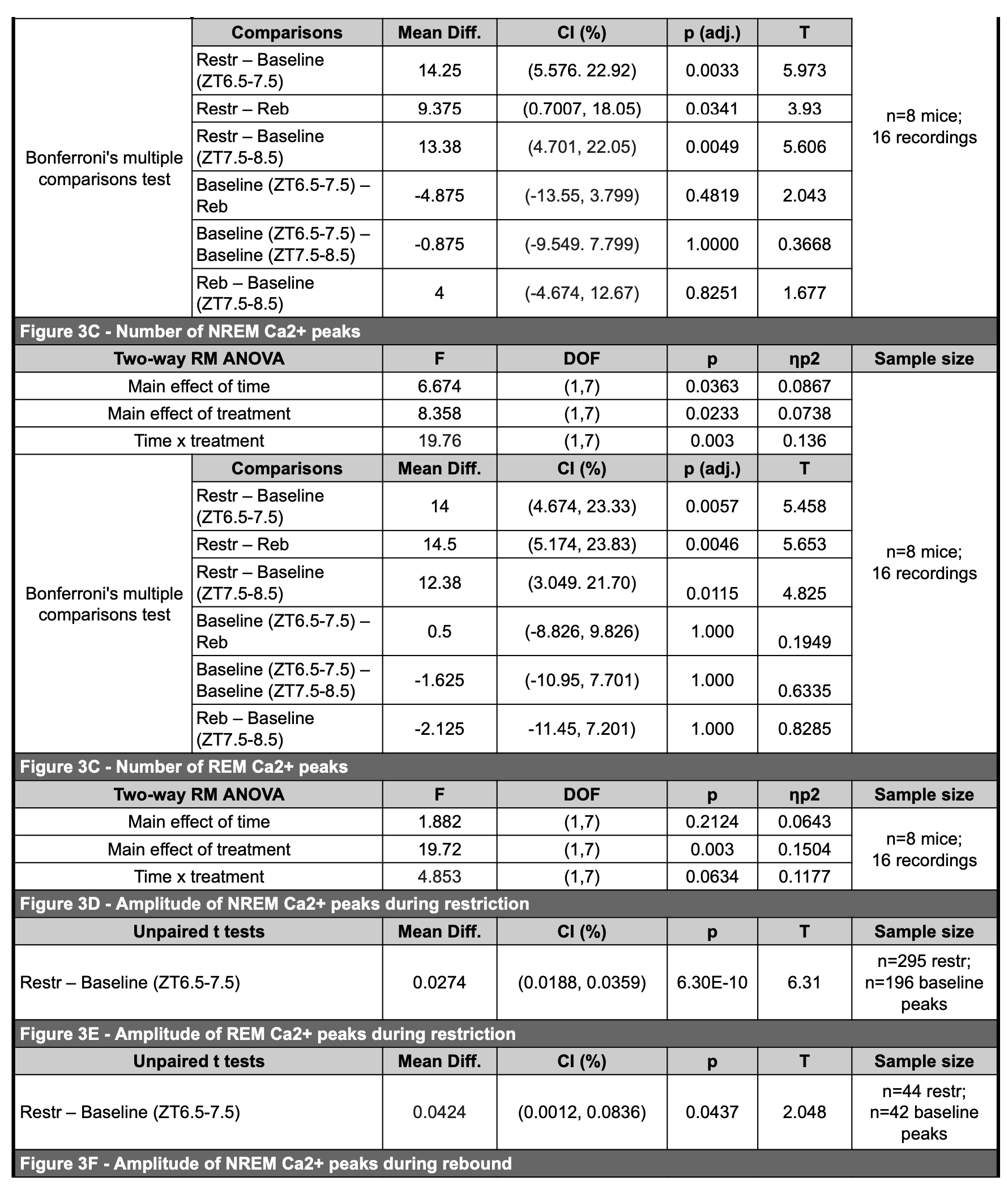


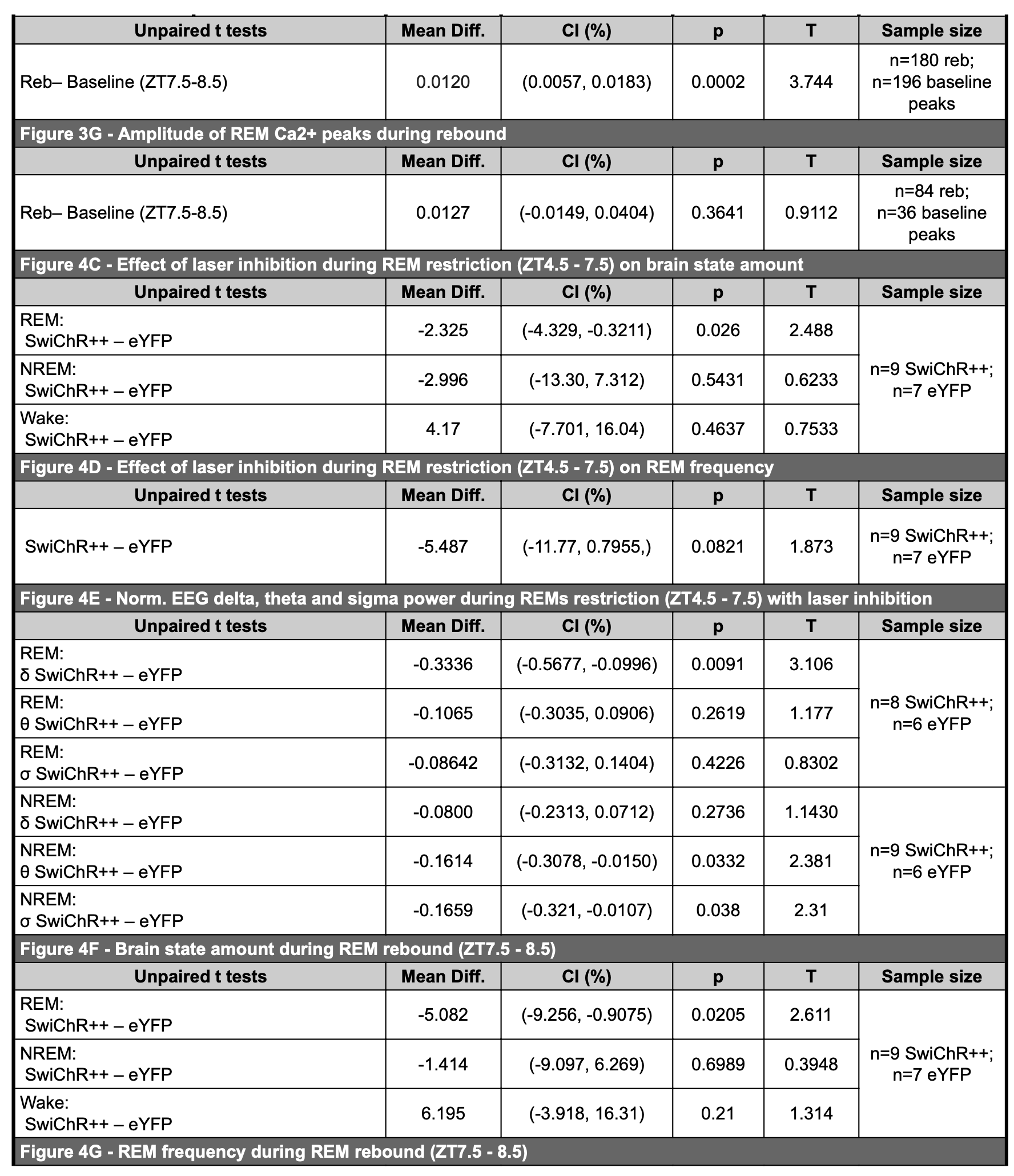


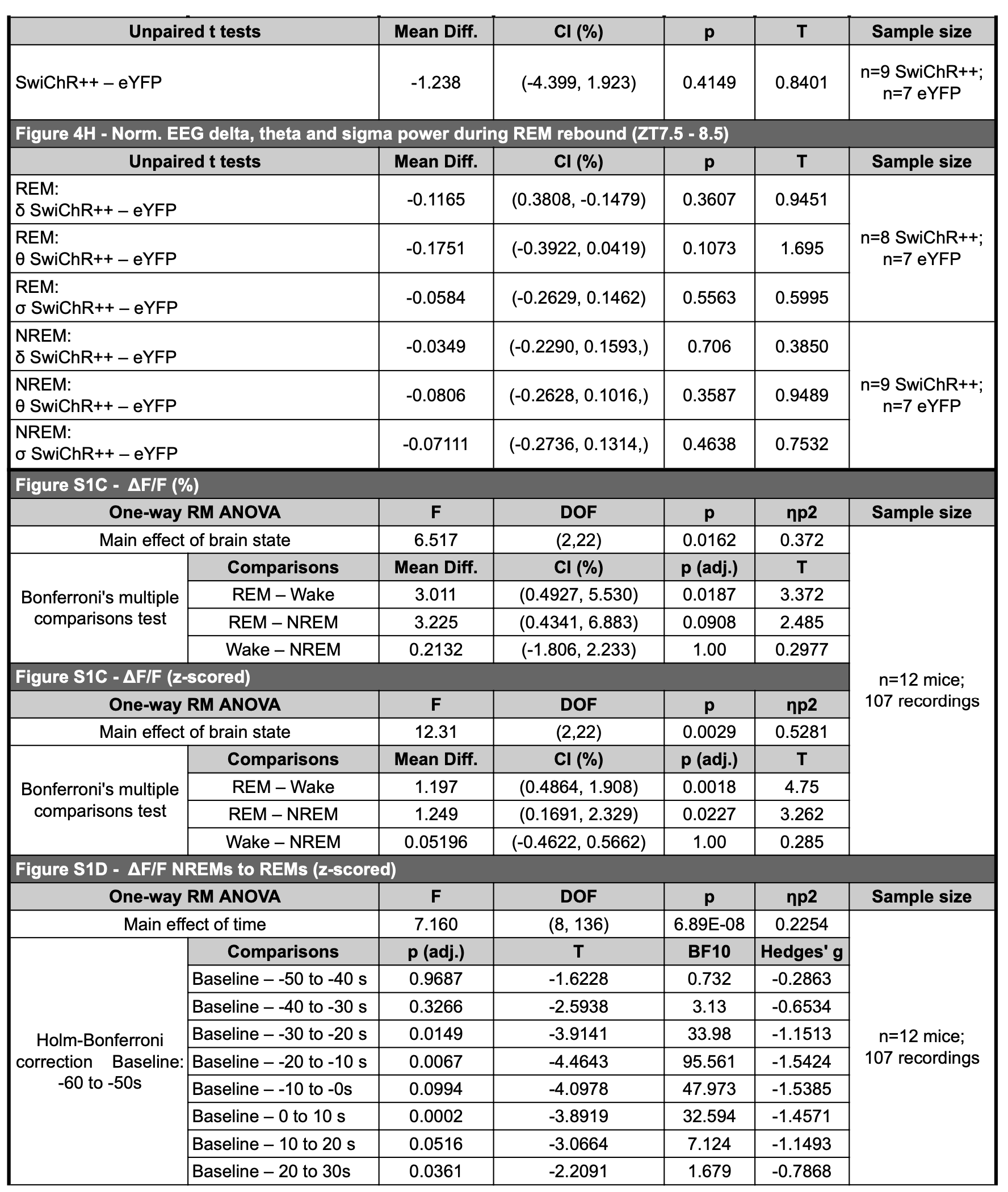


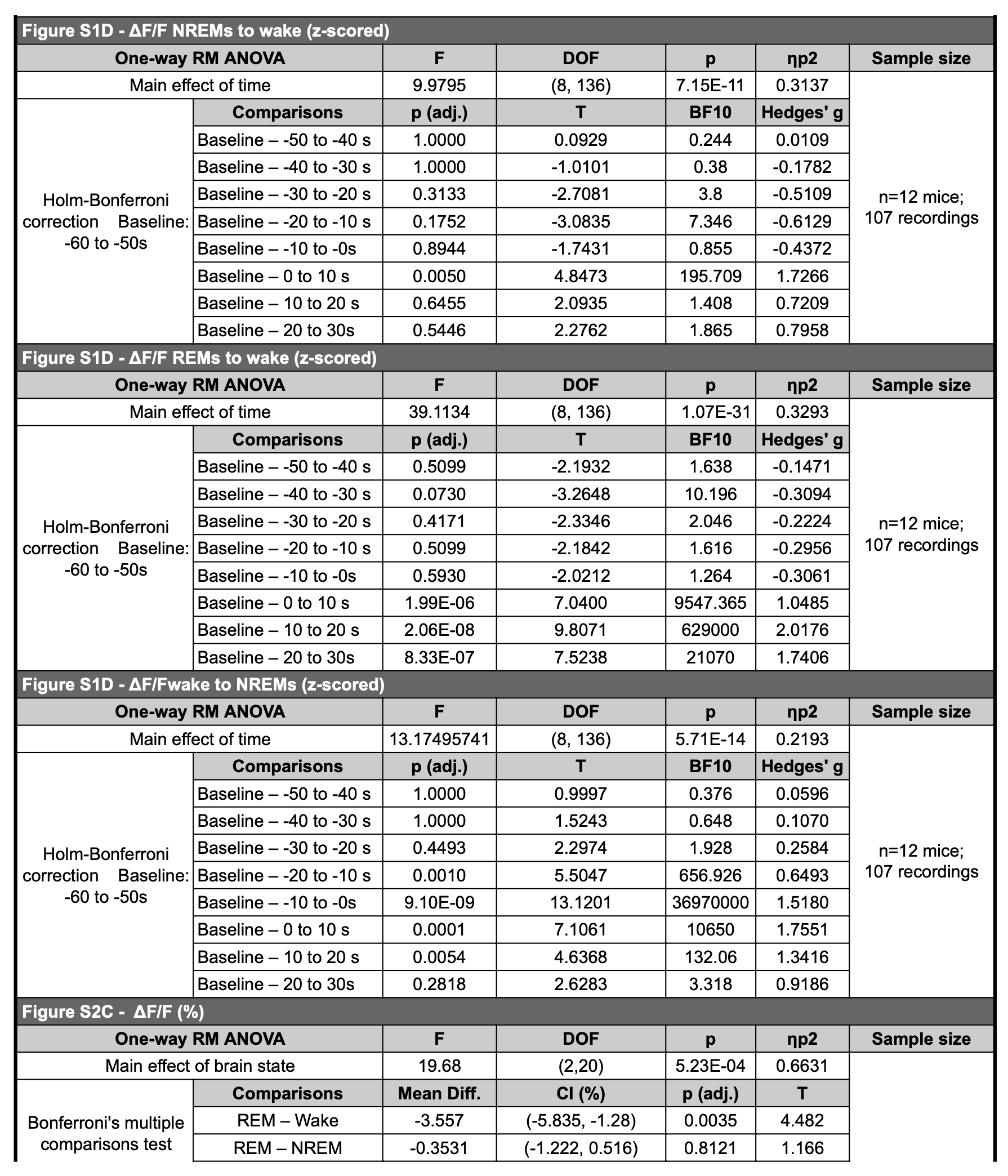


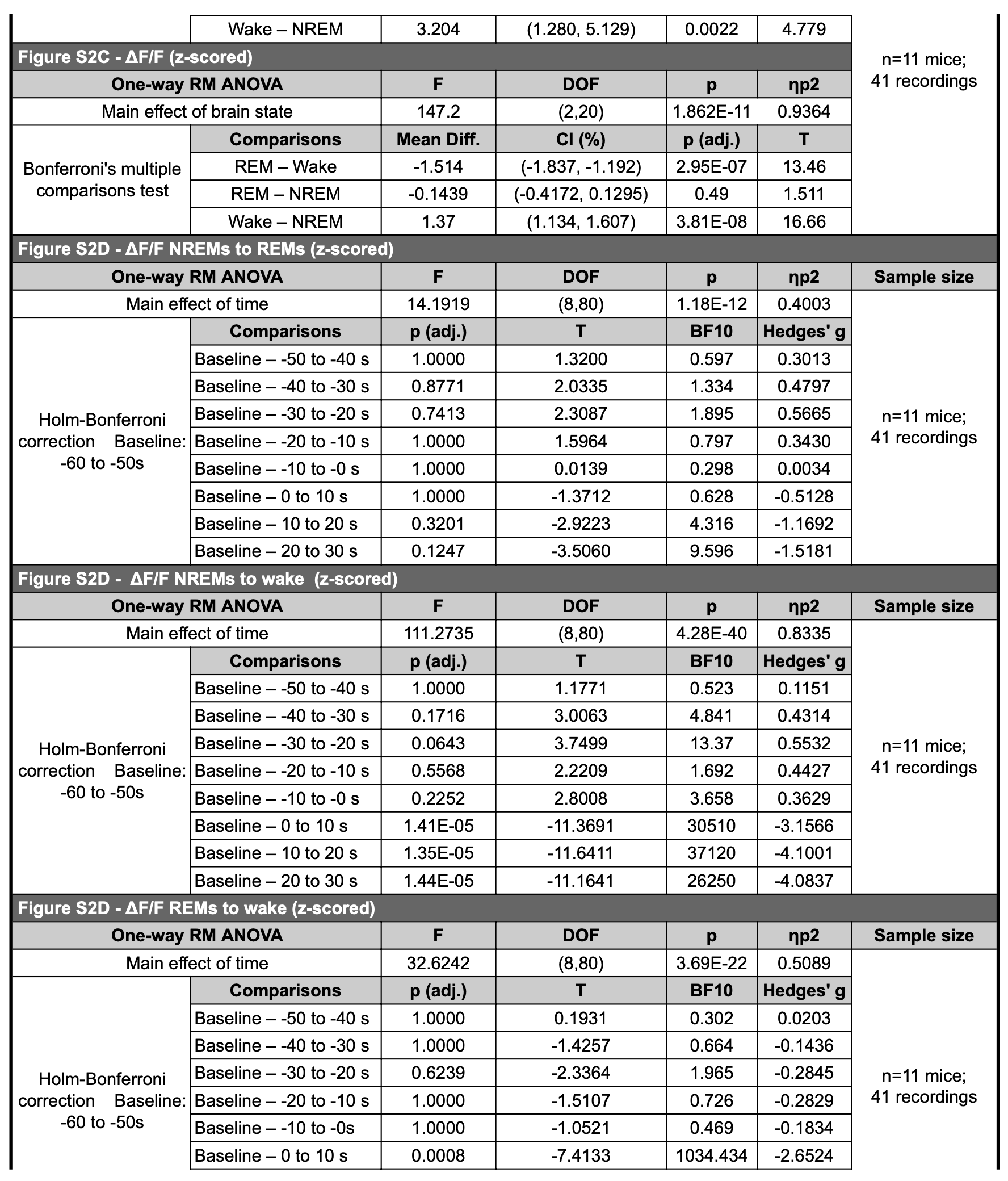


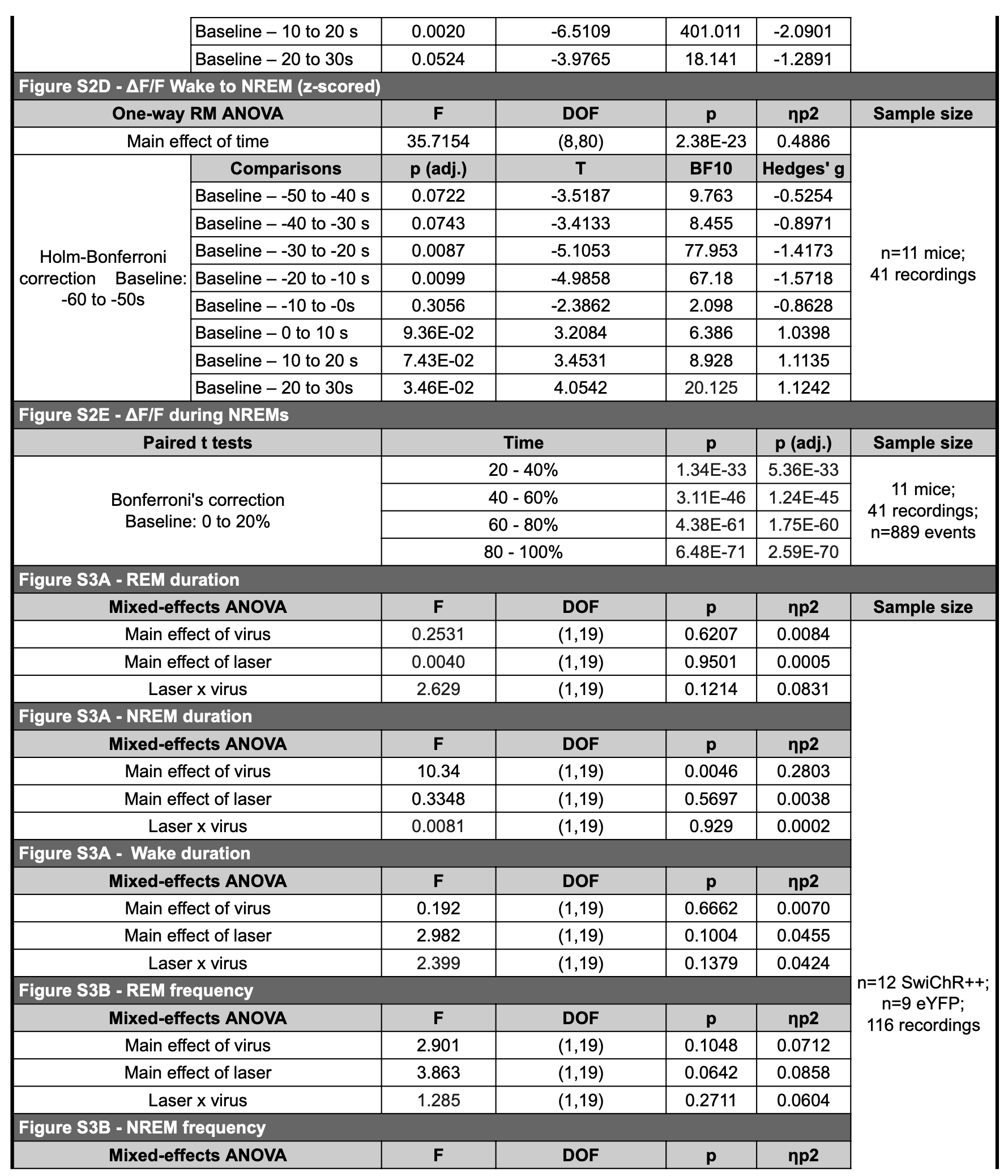


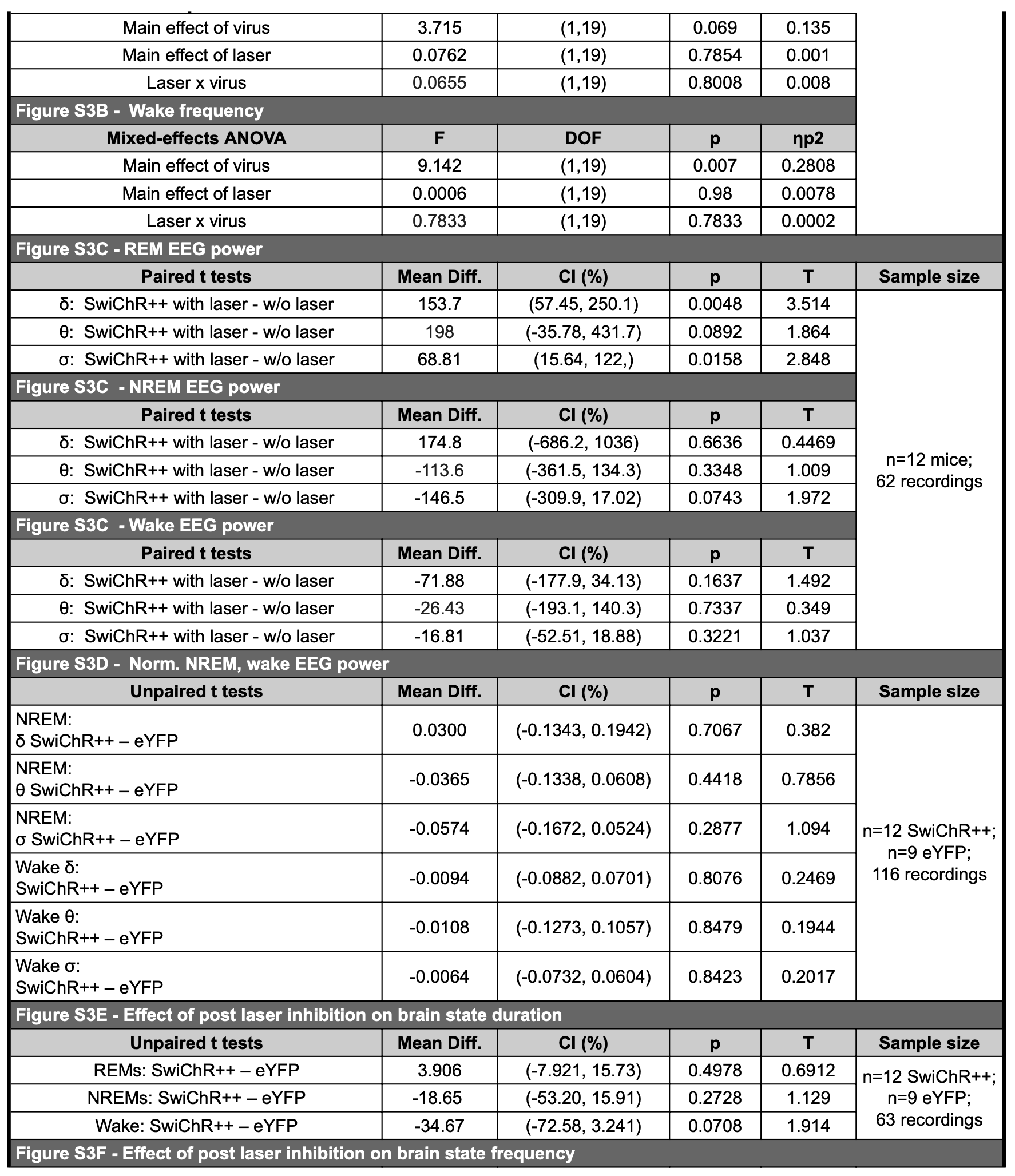


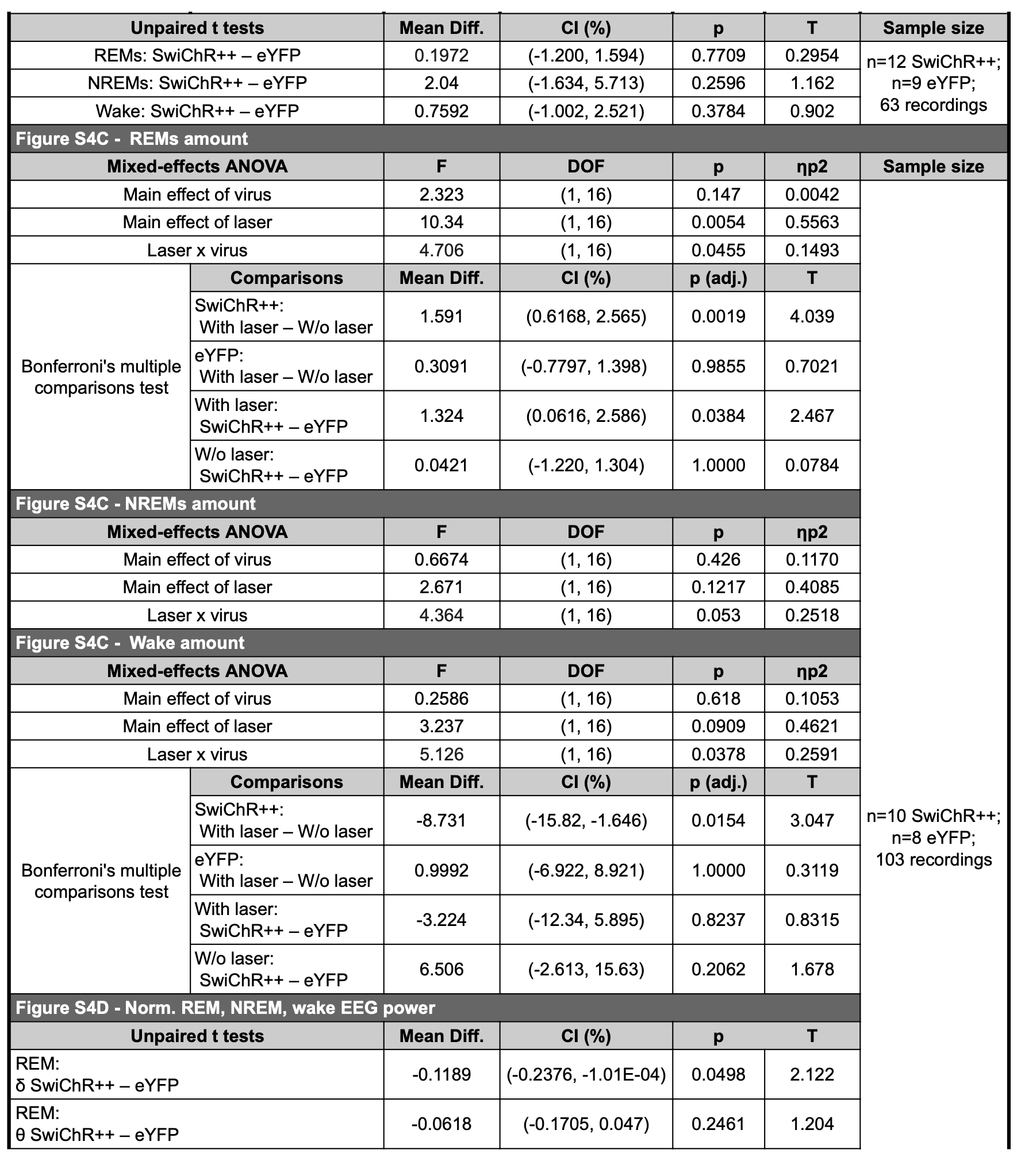


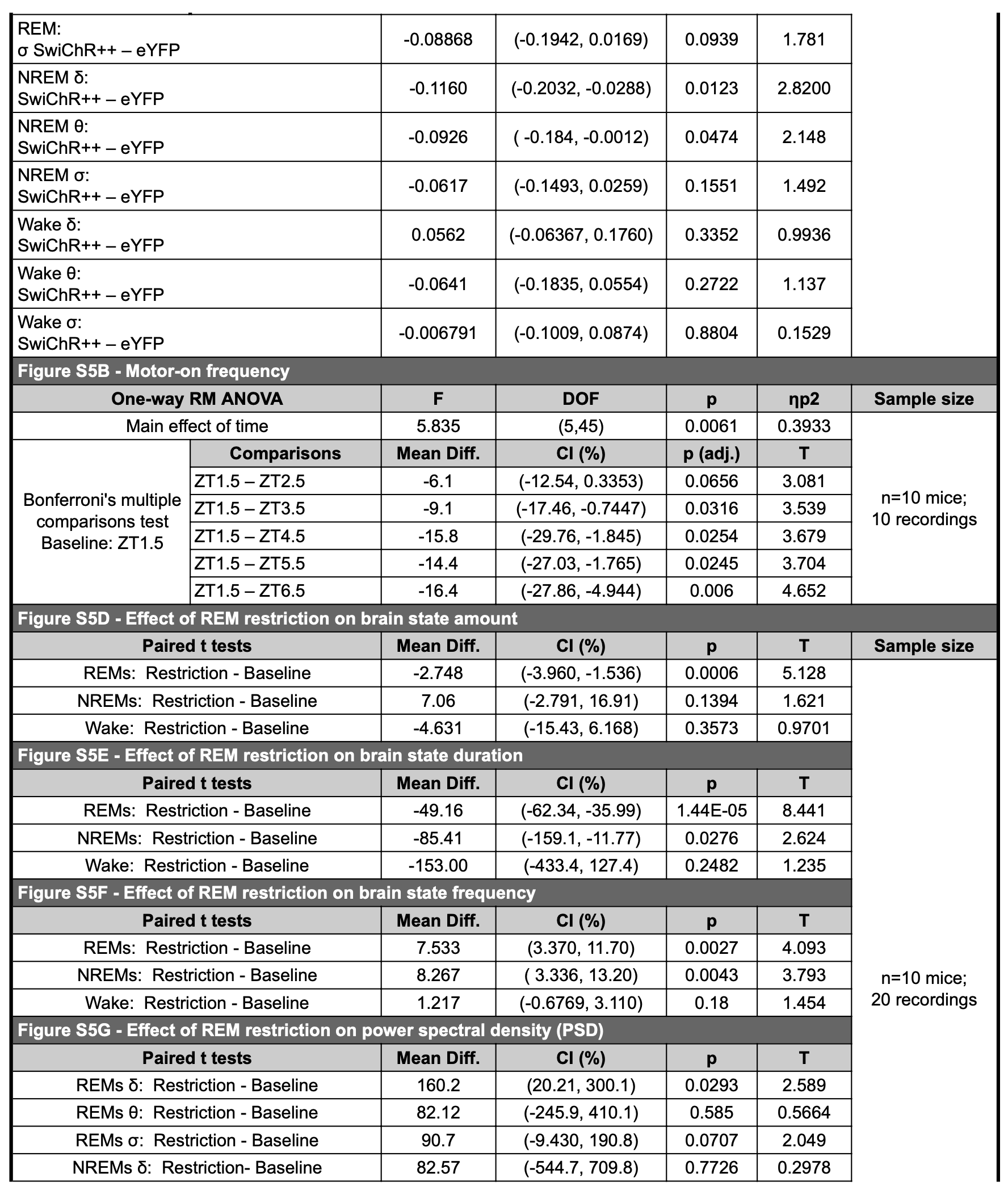


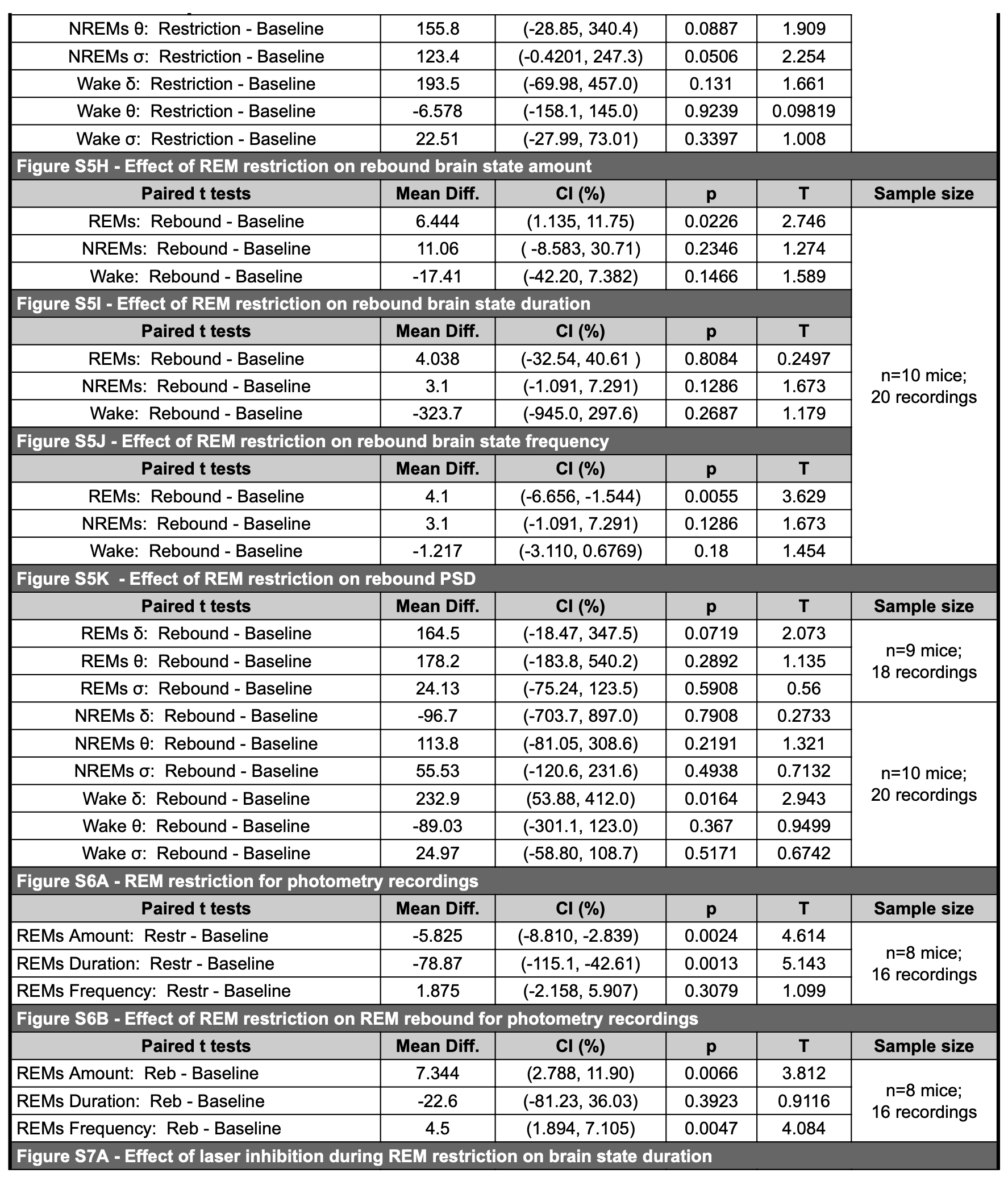


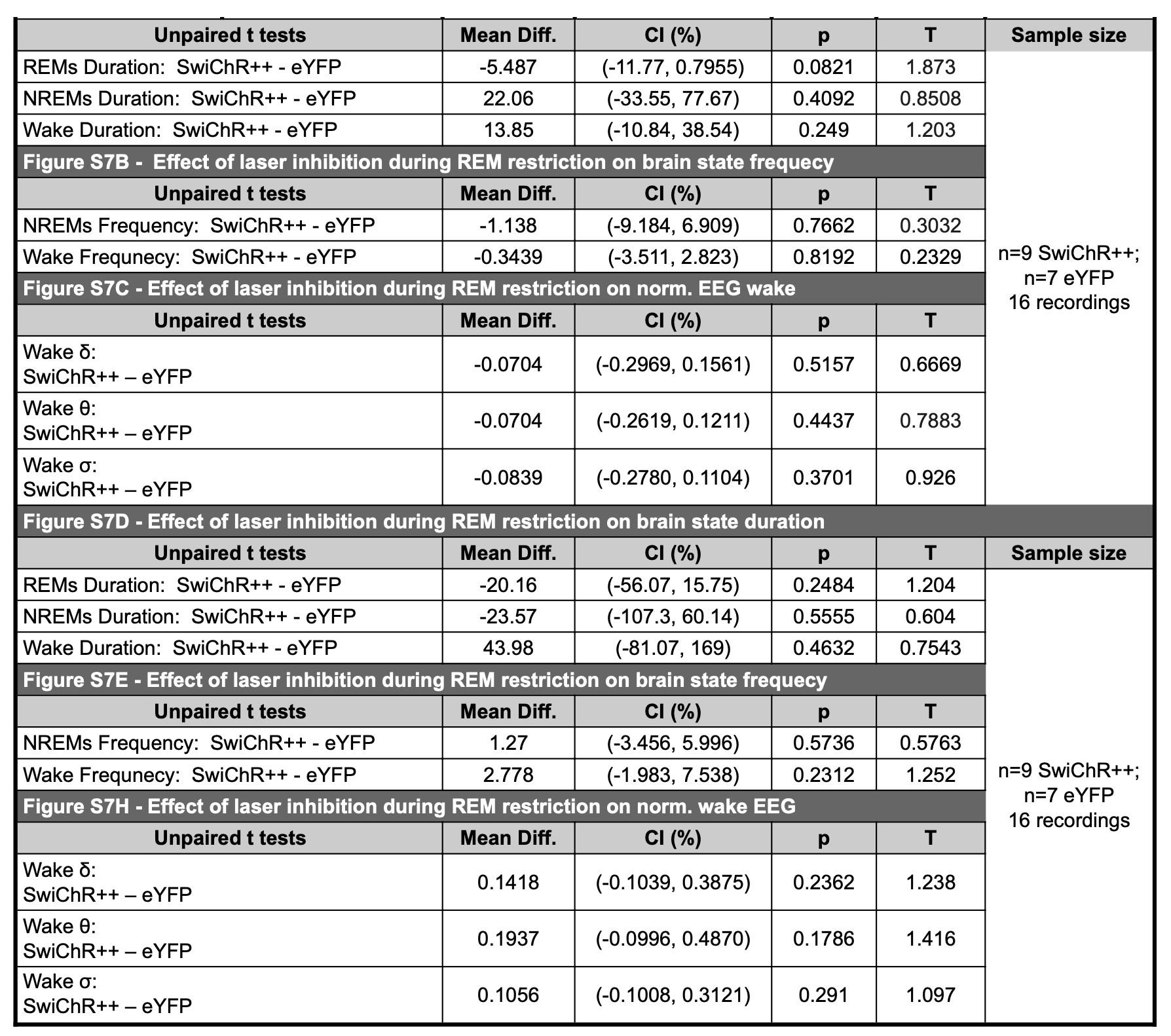

Supplement: Supplementary file 1. [file elife-92095-supp1.docx]
